# Assessment of faculty members’ perceptions towards community-oriented health professions education in Egypt: a concurrent convergent mixed-methods study

**DOI:** 10.1186/s12909-026-09430-1

**Published:** 2026-05-25

**Authors:** Omayma Hamed, Shahinaz Ibrahim Mekheimar, Enjy Abouzeid, Shereen Shaaban Mustafa, Mariam A. Amin, Nezar Abo-Halawa, Nancy Husseiny Hassan, Amira Ebrahim AlSemeh, Wagdy Talaat

**Affiliations:** 1 Medical Education Department, Armed Forces College of Medicine, Heliopolis, Cairo Governorate Egypt; 2 Public Health Department, Theodore Bilharz Research Institute, Cairo Governorate, Egypt; 3 Medical Education Department, Faculty of Medicine, Suez Canal University, Ismailia, Egypt; 4 School of Medicine, Ulster University, Londonderry, UK; 5 Department of Pediatric Dentistry and Public Health, Faculty of Dentistry, Beni-Suef University, Beni-Suef, Egypt; 6 Anatomy Department, Faculty of Medicine, Ain Shams University, Cairo Governorate, Egypt; 7 General Surgery Department, Faculty of Medicine, South Valley University, South Valley, Egypt; 8 Human Anatomy and Embryology Department, Faculty of Medicine, Zagazig University, Sharkia Governorate, Egypt; 9 Egyptian Society for Medical Education (ESME), Ismailia, Egypt; 10 Foundation for Advancement of International Medical Education and Research (FAIMER), Philadelphia, USA

**Keywords:** Faculty perception, Community-oriented health professions education, Mixed-methods study

## Abstract

**Background:**

Despite the growing recognition of Community-Oriented Health Professions Education (COHPE) as an essential framework for developing socially accountable healthcare practitioners, there remains a critical knowledge gap regarding faculty members’ perceptions of COHPE across different health professions disciplines and institutional contexts.

**Aim:**

This study investigates faculty perceptions of COHPE implementation across multiple disciplines and institutions in Egypt.

**Methods:**

A concurrent mixed-methods design was used, involving 454 faculty members from governmental and non-governmental health professions institutions. Quantitative data were collected via validated surveys and analysed using descriptive statistics and agreement indices. Qualitative data from focus group discussions underwent thematic analysis. Integration of both datasets followed a convergent design to identify patterns and contradictions in implementation experiences.

**Results:**

Overall, COHPE implementation was rated as neutral (mean agreement index: 2.8/5), suggesting uncertainty rather than an endorsement issue. Four domains reflected moderate implementation: relevance to community needs, priority health problems, cultural sensitivity, and health systems integration. Domains such as community empowerment, involvement, and partnership development received the lowest scores. Ten overarching themes were identified, with logistical constraints, curriculum rigidity, and faculty development gaps being the most recurrent. Faculty experience did not correlate with stronger COHPE implementation, revealing a paradox in traditional education models.

**Conclusion:**

Findings highlight a widespread conceptual awareness of COHPE principles, but persistent structural and cultural barriers limit their full integration. The convergence of quantitative neutrality and qualitative concern indicates systemic rather than isolated challenges. Strategic reforms targeting curriculum design, faculty training, and stakeholder engagement are essential to transition COHPE from idealistic talk to embedded practice.

**Supplementary Information:**

The online version contains supplementary material available at 10.1186/s12909-026-09430-1.

## Introduction

The evolving landscape of healthcare delivery demands a fundamental transformation in health professions education, shifting from traditional disease-centred approaches to community-oriented frameworks that address population health needs and social determinants of health [1–3]. Community-Oriented Health Professions Education (COHPE) has emerged as a pivotal educational paradigm that aligns health professions curricula with the health needs of communities, fostering socially accountable healthcare practitioners equipped to address diverse health challenges in real-world contexts [4]. This educational approach emphasizes the integration of community perspectives, local health priorities, and population-based interventions into the core curriculum, preparing health professionals to work effectively within complex healthcare systems while addressing health disparities and promoting health equity [5–7].

The theoretical foundations of COHPE are rooted in the principles of social accountability, community engagement, and population health improvement. Unlike conventional medical education models that primarily focus on individual patient care within hospital settings, COHPE adopts a holistic approach that encompasses primary healthcare delivery, community partnership, health promotion, disease prevention, and healthcare system strengthening [6, 8, 9]. This paradigm shift reflects the growing recognition that health outcomes are significantly influenced by social, economic, environmental, and cultural factors that extend beyond the clinical encounter, necessitating healthcare professionals who are competent in addressing these broader determinants of health [7, 10, 11].

Despite the recognized importance of COHPE in producing socially responsive healthcare practitioners, its implementation across health professions education remains inconsistent and challenging [12]. The literature reveals significant variations in how institutions conceptualize, design, and implement community-oriented educational programs, often reflecting differing interpretations of what constitutes effective community engagement in health professions education [13, 14]. Furthermore, the transition from traditional educational models to community-oriented approaches requires substantial institutional commitment, faculty development, curriculum restructuring, and community partnership development, processes that are often hindered by resource constraints, institutional resistance, and lack of clarity regarding implementation strategies [15, 16]. Faculty perceptions and attitudes play a crucial role in the successful adoption and implementation of innovative educational approaches, including COHPE. Recent studies have demonstrated that faculty members’ understanding, acceptance, and enthusiasm for curricular innovations significantly influence their implementation success and sustainability [17]. The challenges associated with implementing community-oriented educational approaches are multifaceted and vary across disciplines. Faculty report significant challenges in conducting self-directed learning, small group discussions, seminars, and tutorials. The perceived barriers also include limited time for teaching health equity, challenges in prioritizing equity content, lack of formal training, discomfort in leading discussions, and institutional barriers such as resource allocation and lack of buy-in. These findings highlight the complexity of faculty engagement with new educational paradigms and underscore the importance of understanding faculty perspectives as a prerequisite for successful curriculum reform. Faculty development needs, resource allocation, institutional support systems, and community partnership establishment represent significant barriers that must be addressed to ensure successful COHPE implementation [18, 19].

The multi-disciplinary nature of health professions education adds another layer of complexity to COHPE implementation. Different health professions, including medicine, dentistry, pharmacy, nursing, and physical therapy, have distinct professional cultures, educational traditions, scope of practice, and community engagement models. These disciplinary differences may influence how faculty members from various health professions perceive and approach community-oriented education, potentially creating disparities in implementation readiness and effectiveness across different programs within the same institution [20, 21]. Recent developments in health professions education have emphasized the importance of interprofessional collaboration and integrated care delivery, concepts that align closely with COHPE principles [22, 23]. However, medical education faces challenges in keeping pace with the rapidly changing healthcare needs of populations worldwide. The COVID-19 pandemic has further highlighted the critical importance of community-oriented health education, as healthcare systems worldwide struggled to address population health needs effectively, revealing gaps in healthcare professionals’ preparation for community-based practice and public health emergencies [24, 25].

While the theoretical benefits of COHPE are well-documented, a significant gap persists in understanding how faculty members across different health professions disciplines perceive this educational approach and the specific challenges they anticipate in its implementation [26, 27]. Much of the existing research is either single-discipline or single-institution focused, limiting generalizability and failing to capture the layered complexity of multi-disciplinary and multi-institutional perspectives on COHPE [28]. This is especially critical given the rising global emphasis on interprofessional education and collaborative practice in healthcare training [10, 22]. Furthermore, the literature often centers on student outcomes and curricular structure, with little exploration of faculty perspectives, particularly their understanding of COHPE’s key determinants and perceived implementation barriers [29]. Addressing this knowledge gap is essential for designing targeted faculty development programs, institutional support mechanisms, and evidence-based implementation strategies suited to diverse educational and healthcare contexts.

Egypt presents a particularly instructive context for examining COHPE implementation. The Egyptian higher education system hosts over 170,000 health professions faculty members distributed across governmental and private universities, overseen by the *Central Agency for Public Mobilisation and Statistics (CAPMAS)*. Despite national health reform agendas that explicitly prioritize primary healthcare and community-based services, the dominant model of health professions education in Egypt remains hospital-centric and discipline-siloed [30–31]. Egypt’s epidemiological profile is characterized by a dual burden of communicable and non-communicable diseases, compounded by significant rural-urban disparities and resource constraints at primary care level. The National Health Insurance Law (Law No. 2 of 2018) has initiated a gradual transition toward universal health coverage, placing renewed pressure on academic institutions to produce graduates capable of community-oriented, preventive, and person-centred care. However, institutional barriers, including rigid centralized curricula, limited faculty development infrastructure, and underdeveloped community partnership mechanisms, have historically constrained meaningful COHPE integration [30, 32]. Against this backdrop, many of the barriers identified in this study, logistical constraints, curriculum rigidity, weak community partnership channels, and faculty resistance, are best understood not as individual lapses but as structural manifestations of an educational system that has *yet to align with community-oriented priorities institutionally.*

### Problem statement

Despite the growing recognition of Community-Oriented Health Professions Education (COHPE) as an essential framework for developing socially accountable healthcare practitioners, there remains a critical knowledge gap regarding faculty members’ perceptions of COHPE across different health professions disciplines and institutional contexts. The lack of a comprehensive understanding of how faculty members from medicine, dentistry, pharmacy, nursing, and physical therapy perceive COHPE principles and implementation challenges limits the development of effective strategies for curriculum reform and faculty development in this area.

This study will be conducted to address the critical knowledge gap in faculty understanding of COHPE across disciplines and institutions. Specifically, it will explore faculty perceptions, anticipated challenges, and context-driven barriers that may influence implementation success. To guide this inquiry, the following research question is posed: *How do faculty members of diverse health professions (medicine*,* dentistry*,* pharmacy*,* nursing*,* and physical therapy) perceive community-oriented health professions education (COHPE) across multiple institutions?*

## Methodology

### Study design

This study employed a concurrent convergent mixed-methods design with a cross-sectional approach to comprehensively assess faculty members’ perceptions of community-oriented health professions education (COHPE). The mixed-methods design was specifically chosen to address the complexity of understanding faculty perceptions, where quantitative data provides measurable insights into perception patterns across disciplines and institutions, while qualitative data offers a deeper understanding of the contextual factors and challenges that influence these perceptions [33]. This methodological approach enables triangulation of findings, enhancing the validity and depth of the research outcomes.

The cross-sectional design allows for efficient data collection across multiple institutions and disciplines at a single time point, providing a snapshot of current faculty perceptions while controlling for temporal variations that might influence responses. Cross-sectional studies are particularly useful for establishing baseline understanding before planning future longitudinal studies [34]. Given the exploratory nature of faculty perceptions toward COHPE, this design is particularly appropriate for establishing a baseline understanding and identifying patterns across diverse academic settings.

### Study setting

The study was conducted across multiple higher education institutions in Egypt, encompassing both governmental and non-governmental universities to ensure comprehensive representation of the country’s health professions education landscape. The multi-institutional approach was deliberately chosen to capture variations in institutional culture, resources, administrative structures, and educational philosophies that may influence faculty perceptions of COHPE. This diversity in institutional contexts enhances the external validity of the findings and their applicability across different educational environments.

Twenty-three institutions participated in the quantitative component, representing a substantial proportion of health professions education providers in Egypt. For the qualitative component, ten institutions were strategically selected to ensure balanced representation across institutional types (governmental vs. non-governmental) and geographic regions, providing rich contextual data that complements the quantitative findings.

### Study population and eligibility criteria

The target population comprised faculty members holding academic positions (lecturers, assistant professors, and full professors) in five health professions disciplines: medicine, dentistry, pharmacy, nursing, and physical therapy. The inclusion of multiple disciplines was essential to capture the interdisciplinary nature of COHPE and understand how different professional backgrounds influence perceptions of community-oriented education.

#### Inclusion criteria

- Full-time faculty members with active teaching responsibilities.
- Academic rank of lecturer or above.
- Affiliation with participating institutions’ health professions programs.
- Willingness to participate in the study.

#### Exclusion criteria

- Part-time or visiting faculty members.
- Administrative staff without teaching responsibilities.
- Faculty members on sabbatical or extended leave during the data collection period.

### Sampling strategy

#### Quantitative component

The sampling approach was designed to balance statistical requirements with practical feasibility constraints. From a total population of 17,010 faculty members across the five disciplines at participating institutions, a strategic sampling approach was implemented based on established psychometric principles for survey research. Following the widely accepted rule of thumb for survey research [35], which recommends 10–15 participants per survey item to ensure adequate statistical power and reliability, our 20-item validated questionnaire required a minimum sample size of 300 participants (15 participants × 20 items). This sample size also aligns with guidelines for cross-sectional studies examining perceptions across multiple groups [36]. The sample was proportionally distributed across participating institutions based on their faculty size to ensure representative coverage. Within each institution, convenient sampling through established academic networks was employed, utilizing institutional WhatsApp groups that included faculty members from each discipline. This strategic sampling approach represents a purposeful methodology that combines the statistical rigor of proportional allocation with the practical efficiency of convenience sampling, an approach that is methodologically justified by the multi-institutional, multi-disciplinary design, which inherently minimizes systematic bias through institutional and disciplinary diversity [37].

#### Qualitative component

To complement the limitations inherent in convenience sampling and enrich the study’s depth, a concurrent qualitative component was strategically embedded in the research design. Ten institutions, representing 43% of the 23 engaged in the quantitative phase, were purposively selected using principles of maximum variation sampling [38]. This approach ensured meaningful representation across: Institutional type **(**governmental vs. non-governmental); geographic distribution (diverse regional coverage); the five included health professions disciplines; and institutional characteristics (varied size and resource availability). This sampling strategy balanced the need for diversity with practical feasibility, allowing for manageable yet robust qualitative insights.

Focus Group Discussions (FGDs) were employed as the primary qualitative data collection method, selected for their capacity to elicit rich, in-depth information and collective reflections on the challenges of implementing COHPE. At each of the ten institutions, two FGDs were conducted (totalling 20 discussions), each comprising 8 participants, a size supported as optimal by Morgan (1997) and Krueger and Casey (2015) [39–40], who recommended 6–12 participants per group for effective engagement. The resulting qualitative sample of 160 participants not only exceeds adequacy benchmarks for saturation in qualitative research [41] but also provides sufficient breadth for cross-institutional and cross-disciplinary thematic analysis.

### Data collection instruments

#### Quantitative instrument

A validated self-administered questionnaire was employed [Appendix 1], anchored in the nine COHPE determinants synthesized from the narrative review of Talaat & Hamed (2024) [7]. The questionnaire comprised:

1. **Demographic section**: Capturing participant characteristics including age, gender, institution type, discipline, academic rank, teaching experience, prior COHPE involvement, and formal training background.
2. **Perception assessment sections**: Nine sections corresponding to COHPE determinants are: (1) Relevance to community needs (2 items); (2) Priority health problems (2 items); (3) Level of integration of community orientation (2 items); (4) Community empowerment and engagement (2 items); (5) Cultural sensitivity and safety (2 items); (6) Social accountability (2 items); (7) Incorporation of health systems science (2 items); (8) Partnering with organizations and government (2 items); and (9) Community involvement in COHPE (4 items).

The survey’s content validation was conducted using the Content Validity Index (CVI) to ensure validity. A panel of nine expert judges who are health professions educationists rated each item on a 4-point scale: 1: Not relevant; 2: Somewhat relevant; 3: Quite relevant; 4: Highly relevant, with consensus defined as ≥ 75% agreement and a median score of 3 or 4 [42–43]. Inter-rater reliability was assessed using the intraclass correlation coefficient (ICC) through ANOVA: Two-factor without replication [44]. The survey was piloted on 20% of the sample, who were excluded from the main study, ensuring the clarity and appropriateness of items. Reliability was measured using Cronbach’s Alpha, with a threshold of ≥ 0.75 considered acceptable.

The (CVI) of 0.875 was achieved, exceeding the recommended threshold of 0.80 [45]. Initially, 21 items were reviewed, and one was removed from Sect. (9) for not meeting the CVI threshold. Reliability analysis yielded a Cronbach’s alpha of 0.89 for the final 20-item version, indicating high internal consistency. The ICC value of 0.865 demonstrated good inter-rater reliability. The pilot study did not reveal any required changes to the questionnaire.

#### Qualitative Instrument

A semi-structured focus group discussion guide was developed [Appendix 2], focusing on challenges faculty members anticipate in implementing COHPE within their curricula. The guide was structured around the nine COHPE determinants, allowing for systematic exploration while maintaining flexibility for emergent themes. The guide was reviewed by the research team and pre-tested to ensure clarity and appropriateness for the target population. The test did not reveal any changes required to the interview guide.

#### Data collection procedures

Quantitative data collection was conducted through an online survey using Google Forms, which was disseminated via institutional academic WhatsApp groups. This strategy was selected for its efficiency in reaching faculty members across different geographic locations and its compatibility with communication platforms commonly used by the target population [46–47]. The digital format also enabled immediate response validation and minimized manual entry errors through automated features such as built-in data checks that permit setting specific rules for each question in the form [48]. To ensure data quality and reliability, the survey included clear instructions and embedded informed consent protocols [33]. Reminder messages were periodically sent to encourage participation, and response patterns were regularly monitored to identify potential issues such as duplicate entries or inconsistent responses.

Qualitative data were collected through face-to-face or Zoom-facilitated focus group discussions held at selected institutions, facilitated by trained co-investigators. Each session lasted approximately 90 to 120 min and was audio-recorded with participants’ consent. The in-person format was deliberately chosen to foster more meaningful interactions and to accommodate preferences for discussing sensitive educational matters in a familiar setting [49–50]. These discussions followed a standardized procedure that included formal introductions, verbal informed consent, and the use of a validated discussion guide aligned with the study’s objectives [50]. This combined approach of digital survey distribution and in-person focus groups allowed for effective triangulation of findings and enhanced methodological rigor across institutional settings [33].

#### Data analysis

Quantitative data were analysed using descriptive statistical methods to summarize participant characteristics and perception scores. The analysis included calculations of frequencies and percentages. To assess the level of consensus among respondents, an agreement index was computed based on the framework outlined by Guilbert in the *Educational Handbook for Health Personnel* published by the World Health Organization [51]. The index was interpreted using the following categories: scores ranging from 1.00 to 2.59 indicated disagreement, 2.60 to 3.39 reflected neutrality, and 3.40 to 5.00 signified agreement.

**Fig. 1:**
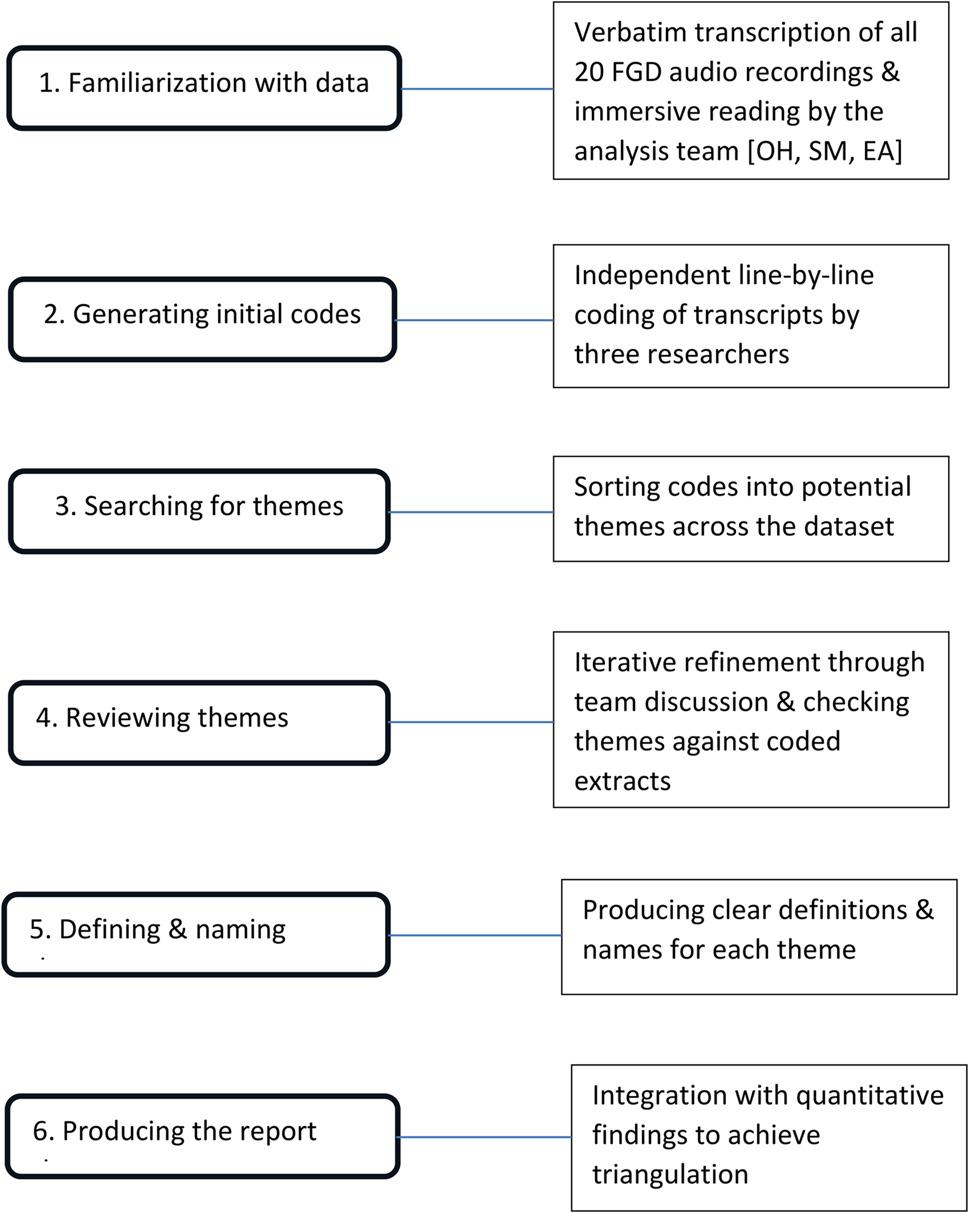
Thematic analysis flowchart according to braun and clarke’s six-phase approach (2006)

Qualitative data from focus group discussions were examined using thematic analysis, following the six-phase approach described by Braun et al. (2006) (Fig. 1) [52]. Audio recordings were transcribed verbatim, and transcripts were independently coded by three researchers [OH-SM-EA]. Preliminary themes were developed through collaborative review and refined iteratively. Final themes were validated through team consensus and integrated with quantitative findings to enrich interpretation and support triangulation.

## Results

### Participant characteristics

A total of 454 faculty members participated in the study, surpassing the target sample size of 300. The participant pool was predominantly female (71%), with the majority employed in governmental institutions (76%). Professors comprised 34% of respondents, and more than half (53%) reported over 15 years of teaching experience. Faculty representation was highest from medicine (57%), followed by pharmacy (15%), dentistry (13%), nursing (9%), and physical therapy (6%), offering a well-rounded disciplinary profile to assess COHPE implementation across institutions (Table 1).

**Table 1:** Participant demographic and professional characteristics (*n* = 454)

Characteristic	Category	Frequency	Percentage (%)
**Gender**	Male	130	29
	Female	324	71
**Institution Type**	Governmental	347	76
	Non-governmental	107	24
**Academic Rank**	Professor	153	34
	Assistant Professor	108	24
	Lecturer	193	43
**Faculty Discipline**	Medicine	261	57
	Pharmacy	70	15
	Dentistry	58	13
	Nursing	39	9
	Physical Therapy	26	6
**Teaching Experience**	< 5 years	59	13
	5–10 years	76	17
	11–15 years	78	17
	> 15 years	241	53

The heat map in Table 2 presents faculty perceptions across the nine COHPE domains, with most scores landing in the “neutral zone” (AI: 2.6–3.39). This range reflects a state of implementation vagueness, whereby faculty responses reveal uncertainty or mixed experiences rather than strong confidence in how well COHPE principles are embedded in the curriculum. Four domains demonstrated moderate levels of implementation: *Relevance to Community Needs* (AI: 3.0), *Priority Health Problems* (AI: 2.9), *Cultural Sensitivity and Safety* (AI: 2.9), and *Health Systems Science Integration* (AI: 2.95). Faculty generally affirmed curricular relevance, with over 83% providing neutral to positive responses; however, the qualitative data pointed to curriculum rigidity. As one faculty member noted, *“The curriculum is too packed to add anything new*,*” (FM-Inst.7)* echoing a common logistical constraint that obstructs reform. Curricular coverage of major health problems was acknowledged by 78% of faculty, while qualitative themes highlighted data deficiencies and needs assessment challenges as critical barriers. Specifically, participants from multiple institutions flagged data access and prioritization as a serious impediment: *“Lack of accessible*,* representable*,* reliable*,* and accurate data/statistics on health problems of the community*,* especially in Egypt”* (FM-Inst.1); and *“There is no continuously updated community health needs assessment”* (FM-Inst.2). Moreover, gaps in cultural training revealed critical blind spots in responsiveness. While cultural sensitivity received average scores, faculty acknowledged they often lacked the skills or confidence to address diverse community contexts appropriately; a signal of unmet faculty development needs. This was captured in remarks such as *“Lack of training in dealing with different cultures and situations”* (FM-Inst.3) and *“Lack of specialized faculty to train faculty members in this aspect as regards: content*,* objectives*,* teaching*,* and assessment methods”* (FM-Inst.10). Integration of health systems science was also constrained by overloaded curricula and limited expertise: *“HSS spans multiple disciplines*,* requiring collaboration across departments and integration of diverse perspectives*,* which can be logistically complex”* (FM-Inst.4), further illustrating the multi-faceted challenges behind even “moderate” implementation levels.

**Table 2:**
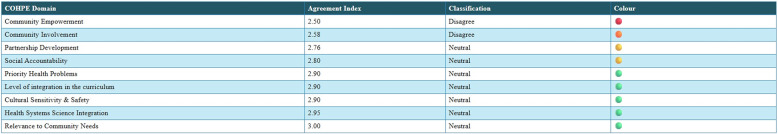
Heat map showing the agreement indices across COHPE domains

This is a colour gradient heat map showing agreement index per domain, with red representing low agreement and green representing higher neutrality

In contrast, lower scores emerged in domains emphasizing community collaboration: *Community Empowerment and Engagement* (AI: 2.5), *Community Involvement in COHPE* (AI: 2.58), and *Partnership Development* (AI: 2.76). These numbers reflect weak agreement from faculty regarding community participation in curriculum design. Only 17.6% affirmed active involvement, pointing to a disconnect between academic structures and community stakeholders. Qualitative findings reinforced this through themes of weak communication channels and a lack of strategic planning frameworks. One respondent observed, *“We rarely involve community voices unless there’s a project requirement*,*”* (FM-Inst.1), illustrating the limited and episodic nature of engagement. Other participants described structural barriers to community access: *“No channels of connections between the community and educational members”* (FM-Inst.5), and *“Dissociation between university and community-lack of support-resistance of both staff member and community leader to change the classical way of teaching and dealing with the community needs”* (FM-Inst.6). The theme of community distrust also recurred across institutions: *“Lack of interest and mistrust health professionals”* (FM-Inst.7), and *“Closed communities that refuse to interact or respond to educators”* (FM-Inst.8). The lowest agreement indices were observed in *Community Empowerment* and *Community Involvement*, both falling below 2.6. Qualitative responses emphasized community mistrust and lack of engagement, exemplified by the quote: “*Communities don’t trust our intentions.”* (FM-Inst.2). Faculty consistently described ineffective partnership channels, emphasized by the qualitative themes of administrative barriers and weak institutional commitment. Participants highlighted bureaucratic obstruction as the primary impediment to sustained collaboration: *“Bureaucratic Procedures and Administrative Delays”* and *“Lack of commitment and continuity work of both sides”* (FM-Inst.7). Partnership with government bodies was further impeded by security requirements in some institutions: *“Partnerships require a lot of approvals from military bodies for security reasons”* (FM-Inst.1). Regarding social accountability more broadly, participants noted the gap between concept recognition and practice: *“The problem is in the practical application of this social accountability”* (FM-Inst.8), while others confirmed institutional resistance: *“Faculty and administrators may resist altering traditional curricula*,* fearing disruption or uncertainty about the benefits of socially accountable practices”* (FM-Inst.9). [Appendix 3] shows the detailed agreement frequencies.

**Fig. 2:**
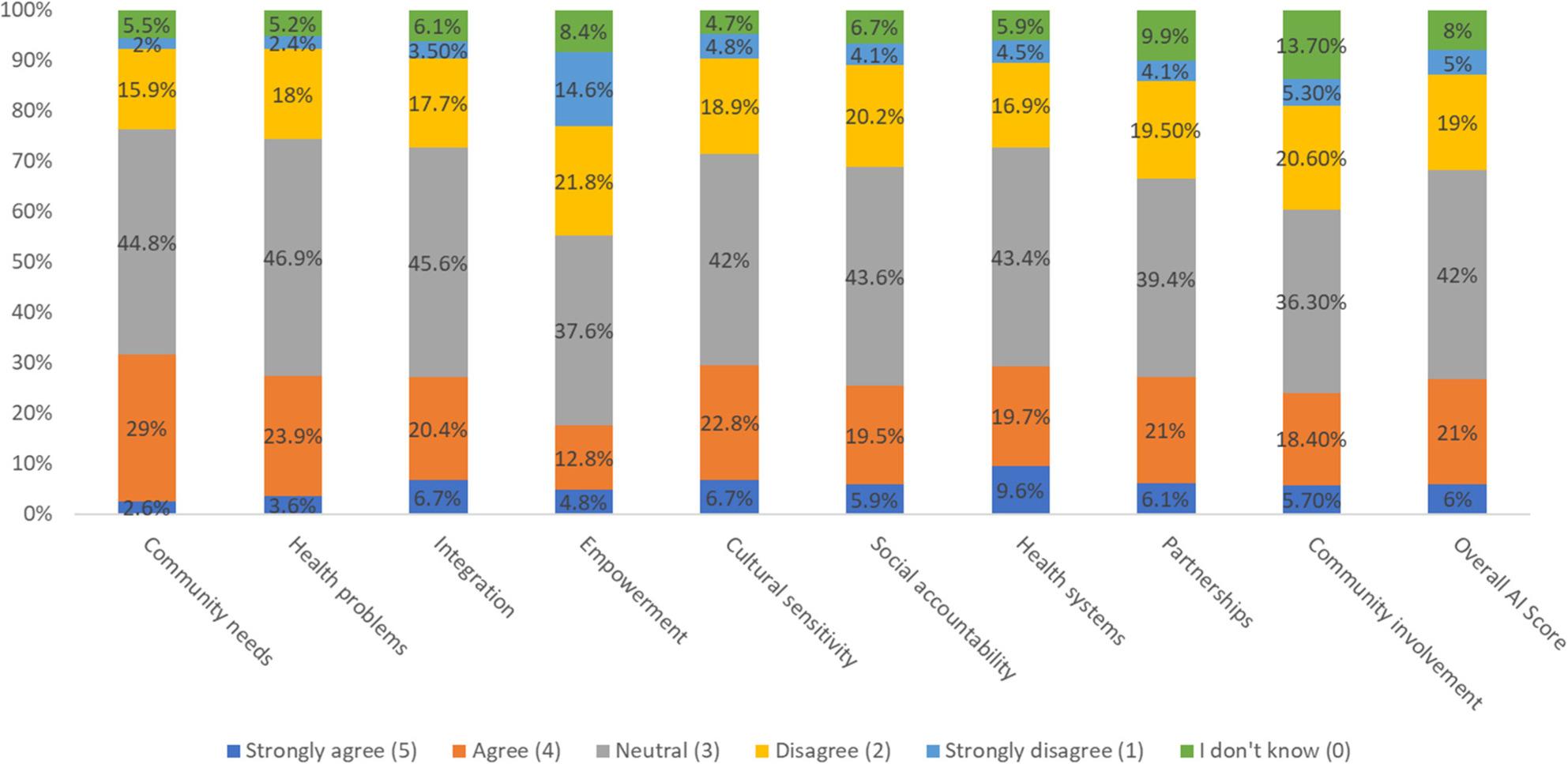
Response distribution by COHPE domain

Figure 2 shows the distribution of faculty responses across all nine COHPE domains using a six-point Likert scale. Across most domains, the majority of participants reported neutral or mildly positive perceptions, reinforcing the earlier finding of implementation uncertainty. For example, responses to *Community Needs* and *Health Problems* skewed toward neutrality (44.8% and 46.9%, respectively), with moderate levels of agreement suggesting partial curricular alignment. Domains such as *Empowerment* and *Community Involvement* showed elevated levels of disagreement (21.8% and 21% respectively), indicating weaker engagement practices and broader concerns around community partnership integration. The proportion of strong agreement remained low across all domains, while the presence of both disagreement and neutrality in the *Overall AI Score*, where 42% were neutral and 34.7% disagreed, further reflects the mixed perception of COHPE implementation among respondents. This distribution highlights key areas for focused improvement, particularly in domains where consensus and confidence are lacking. [Appendix 3]

To explore systemic barriers, Table 3 shows how qualitative themes were mapped across COHPE determinants. The matrix highlights how faculty challenges transcend individual domains and point to deeper, institutional-level limitations. Nearly all themes appeared in a majority of domains, confirming structural consistency.

**Table 3:** Theme frequency distribution matrix across determinants

Determinants (D)	D1	D2	D3	D4	D5	D6	D7	D8	D9	Frequency
Common Themes										
Logistical constraints	x	x	x	x	x	x	x	x	x	9/9
Faculty development gaps	x	x	x	x	x	x	x	x	x	9/9
Faculty resistance	x	x	x	x	x	x	x	x	x	9/9
Organizational Leadership	x	x	x		x	x	x	x	x	8/9
Policy/Implementation Barriers	x	x	x	x	x	x	x	x		8/9
Data/Assessment Gaps	x	x		x	x	x	x	x	x	8/9
Curriculum misalignment	x	x	x		x	x	x			6/9
Communication Gaps	x	x	x	x				x	x	6/9
Community Engagement Issues	x	x	x	x	x	x				6/9
Perceived irrelevance	x	x							x	3/9

Table 4 shows a notable observation that domains 4 and 9 (*Community Empowerment* and *Involvement*) showed both the lowest AI scores and strongest qualitative concerns, making them high-priority areas for strategic intervention. The clustering of domains in the “neutral zone” suggests faculty uncertainty rather than resistance to the idea, signalling opportunities for structural reform and faculty training.

**Table 4:** Convergent validity between quantitative findings and common qualitative themes

Determinant Domain	AI Score	Quantitative Interpretation (Agreement Level)	Dominant Qualitative Themes	Convergence	Integration Interpretation
**Relevance to Community Needs**	3.00	Neutral	- Logistical barriers - Curriculum rigidity	✅ Strong	Moderate awareness, significant barriers
**Priority Health Problems**	2.90	Neutral	- Data deficiencies - Needs assessment gaps	✅ Strong	Recognition of the problem without actionable implementation
**Level of Integration**	2.90	Neutral	- Faculty coordination issues - Limited alignment of curriculum components	🔄 Moderate	Partial integration with visible structural gaps
**Community Empowerment**	2.50	**Disagree**	- Communication gaps - Exclusion from planning	✅ **Strong**	Clear implementation failure
**Cultural Sensitivity**	2.90	Neutral	- Curriculum gaps - Faculty unpreparedness - Training needs	🔄 Moderate	Awareness exists without actionable implementation
**Social Accountability**	2.80	Neutral	- Curriculum limitations & rigidity - Faculty resistance	✅ Strong	Concept recognition: Practice barriers
**Health Systems Integration**	2.95	Neutral	- Overloaded curriculum - Expertise gaps	🔄 Moderate	Structural constraints inhibit adoption.
**Partnership Development**	2.76	Neutral	- Bureaucratic barriers - Weak commitment	✅ Strong	Systemic partnership challenges & collaboration failure
**Community Involvement**	2.58	**Disagree**	- Strategic planning gaps - Trust issues	✅ **Strong**	Fundamental engagement failure; Structural absence of inclusion

**Fig. 3:**
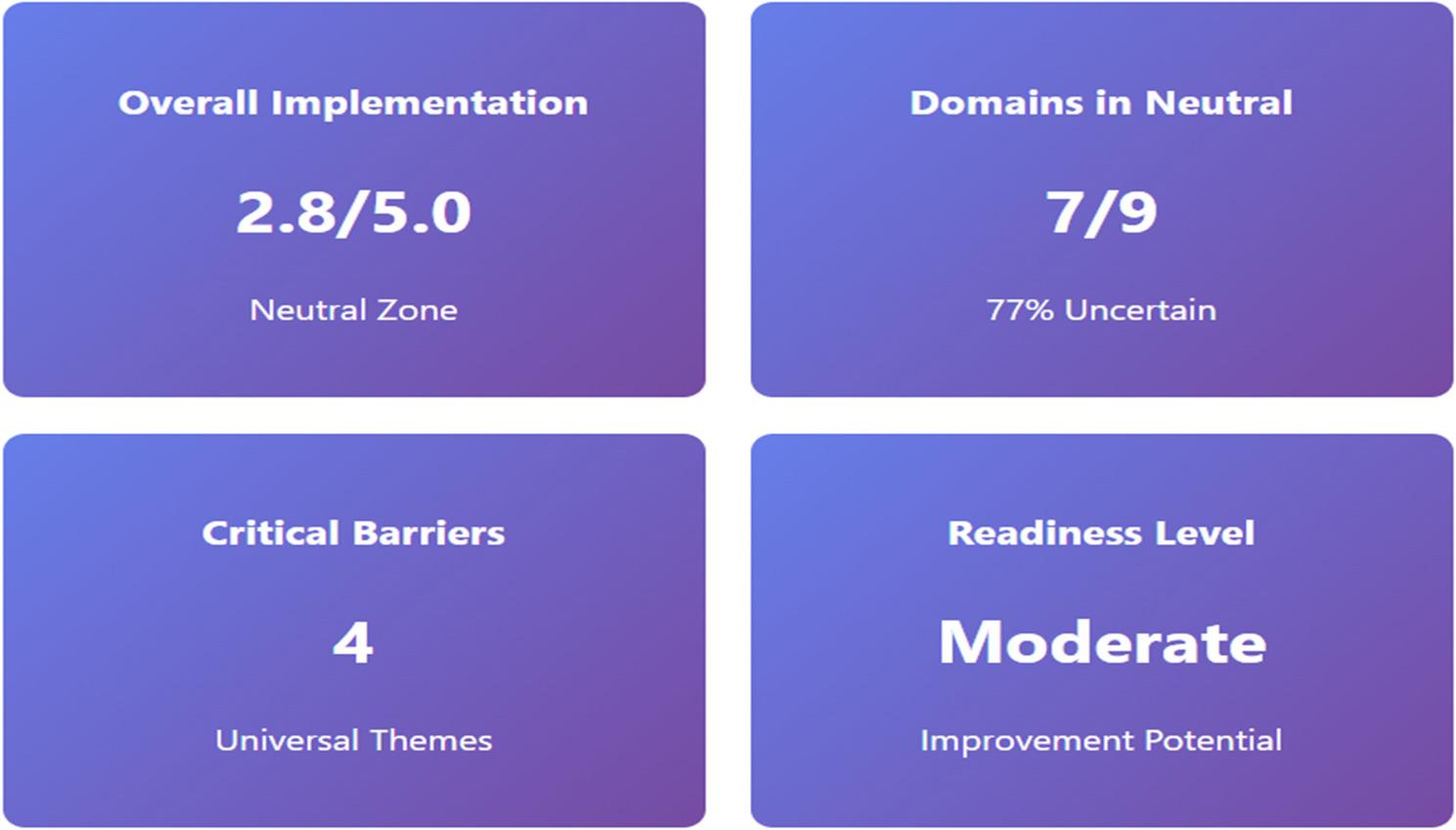
COHPE Implementation readiness infographic

Figure 3 shows an infographic that presents an integrated snapshot of faculty perceptions regarding the implementation status of (COHPE) across participating institutions. The overall implementation score was 2.8 out of 5.0, placing COHPE within the *neutral zone*, suggesting that faculty neither fully endorse nor reject current practices, but rather express uncertainty or mixed experiences, as previously noted in Tables 2 and 4. Of the nine evaluated domains, seven (77%) fell within the neutral zone, confirming that ambiguity predominates across key curriculum components. This wide clustering points to partial awareness and fragmented implementation rather than a consolidated systemic success. The dashboard identifies four universal barriers: logistical constraints, faculty development gaps, curriculum misalignment, and systemic resistance, as persistent across all domains, reinforcing the interpretation that challenges are structural and not isolated to particular disciplines or institutions. The implementation readiness level could be classified as “Moderate,” reflecting a promising foundation for reform. While substantial barriers remain, faculty responses indicate openness to improvement and suggest that with strategic interventions, institutions may transition from passive awareness to active engagement with COHPE principles. This readiness profile supports the rationale for targeted faculty development and institutional restructuring to enhance relevance, responsiveness, and sustainability of community-oriented education.

## Discussion

This comprehensive mixed-methods study provides critical insights into faculty perceptions of Community-Oriented Health Professions Education (COHPE) across multiple disciplines and institutions in Egypt.

### Faculty agreement index

The findings reveal a complex landscape where moderate awareness exists alongside significant implementation barriers, highlighting the urgent need for systematic and strategic approaches to COHPE integration in health professions education. The overall neutral agreement index (2.8/5) observed across COHPE domains suggests that faculty members neither strongly embrace nor reject community-oriented approaches, indicating a state of implementation uncertainty rather than active resistance. Such neutrality presents both a challenge and an opportunity for reform initiatives. This finding aligns with a study by Noya et al. (2024) [53] that identified persistent tensions in embedding social accountability despite institutional endorsement. They found that medical education institutions struggle with integrating social accountability into undergraduate medical education despite recognizing its importance. Domains related to community empowerment (AI: 2.5) and community involvement (AI: 2.58) were the lowest scoring, reflecting systemic barriers such as rigid curricula, misaligned pedagogy, and disciplinary silos. These findings echo global concerns about the difficulty of operationalizing authentic community partnerships in health professions education [54–56]. The convergence of quantitative scores and qualitative themes provides robust evidence of fundamental gaps in community-centred approaches.

### Systemic barriers and implementation challenges

The identification of ten overarching themes through integrated analysis provides a comprehensive understanding of the multifaceted challenges facing COHPE implementation. The universality of “Logistical Constraints,” “Faculty Development Gaps,” and “Faculty Resistance to Change” across nearly all domains (9/9 domains) indicates that these are not isolated issues but systemic challenges requiring institutional-level interventions. Similar constraints have been documented in the implementation of competency-based education, highlighting faculty struggles with restructuring, assessment, and resources [57–58]. The prevalence of logistical constraints reflects the resource-intensive nature of community-oriented education, which requires significant investments in community partnerships, transportation, supervision, and infrastructure. The agreement among participants that the inability to appoint additional assistant professors represents a significant challenge further underscores the human resource constraints facing institutions. This finding is consistent with Soemantri et al. (2018), who highlighted resource allocation as a primary barrier to innovative educational approaches [19]. Faculty development gaps emerge as equally critical, with faculty reporting significant challenges in conducting self-directed learning and small group discussions. This finding suggests that the pedagogical shift required for COHPE implementation extends beyond content knowledge to encompass new teaching methodologies and community engagement skills. The need for comprehensive faculty development programs that address both content expertise and pedagogical competencies becomes apparent from these findings [58].

The universality of these barriers across disciplines further suggests that institutional rather than individual solutions are required. To better understand these barriers, the study draws on the Consolidated Framework for Implementation Research (CFIR), which provides a comprehensive taxonomy of constructs across five domains: intervention characteristics, outer setting, inner setting, characteristics of individuals, and implementation process [30]. The CFIR emphasizes the importance of contextual determinants, including equity and stakeholder engagement, which were reflected in faculty narratives in the FGDs describing mistrust, poor communication channels, and a lack of strategic frameworks for collaboration. These insights reinforce the need to center innovation and equity determinants in COHPE implementation strategies.

### Faculty experience and competency disconnect

Despite over half of respondents (53%) having more than 15 years of teaching experience, this did not correlate with higher COHPE implementation, an insight we term the faculty experience paradox. This supports findings by Woollard & Boelen (2012), who emphasize that traditional teaching experience does not automatically translate to community-oriented competency and highlight the necessity of formal training beyond tenure, and that achieving social accountability in curricula demands purposeful faculty development tied to community priorities. The emphasis should be on transforming experienced educators into community-engaged practitioners rather than assuming that teaching experience alone provides adequate preparation for COHPE implementation [60].

Hence, the pervasive nature of faculty development gaps across all COHPE domains underscores the need for comprehensive capacity-building initiatives. Faculty require support not only in understanding COHPE principles but also in developing practical skills for community engagement, cultural competency, and social accountability [60]. Steinert et al. (2025) further advocate the importance of competency-based frameworks and longitudinal faculty development [58].

### Disciplinary and institutional variations

The study’s multi-disciplinary approach revealed important variations in COHPE perceptions. With medicine comprising 57% of participants, followed by pharmacy (15%) and dentistry (13%), the findings reflect the dominance of medical education in health professions research. However, the inclusion of nursing and physical therapy provides valuable insights into interdisciplinary perspectives, which are increasingly emphasized in integrated learning models [61]. The predominance of governmental institutions (76%) reflects Egypt’s higher education landscape and highlights resource constraints, bureaucratic inertia, and regulatory influences that affect COHPE uptake and implementation. Similar challenges in the Eastern Mediterranean have been outlined by Abdalla et al. (2022) and PAHO (2023), emphasizing the disconnect between accreditation and social accountability [30, 32]. This is echoed by Saniee et al. (2025), who stated that accreditation standards and regulatory frameworks often hinder progress toward social accountability [62].

### Community engagement paradox

The study reveals a concerning paradox in community engagement, where faculty acknowledge the importance of community involvement but struggle with practical implementation. The low scores for community empowerment and engagement domains, combined with qualitative themes highlighting communication gaps and perceived community resistance, suggest a fundamental disconnect between educational institutions and the communities they aim to serve. The lack of community involvement in curriculum development (only 17.6% agreement) represents a significant departure from COHPE principles and suggests that current educational approaches may not adequately reflect community health priorities and needs. Thus, the study’s findings regarding community engagement reflect broader global concerns. Themes of communication gaps, trust deficits, and strategic planning failures align with recent literature emphasizing that social accountability requires health professionals who understand social determinants of health and promote community well-being [63–64]. The weak performance in community empowerment domains suggests that current educational approaches may be maintaining paternalistic models rather than fostering genuine partnerships [65–66].

#### The need for structural reform of curricula

Curriculum misalignment requires structural reform, not just incremental adjustments. This includes revisiting pedagogy, assessment, and learning environments to reflect real-world health needs. Restini et al. (2024) argue for service-learning and outreach integration to elevate community wellbeing [67]. Callahan et al. (2020) and Palsdottir et al. (2016) similarly emphasize aligning curricula with social and economic realities facing communities [68–69]. This aligns with contemporary discussions about the need for transformative changes in health professions education that go beyond incremental modifications [61].

## Conclusion and future research

Faculty perceptions of COHPE in Egyptian institutions reflect moderate conceptual awareness but limited operational integration. The convergence of quantitative neutrality and qualitative concern underscores the need for strategic, systemic reform. Without addressing structural and cultural barriers, COHPE risks remaining an idealistic talk rather than a transformative educational paradigm.

Future research should investigate student perceptions and readiness for COHPE to complement faculty insights; evaluate the impact of faculty development programs on COHPE competencies; and conduct comparative studies across regional contexts to understand local influences on COHPE implementation.

### Limitations of the study

The use of convenience sampling was counterbalanced by engaging a large number of participating institutions and incorporating a concurrent qualitative component, which provided richer contextual insight. Although the study employed a cross-sectional design with data collected at a single time point, this was mitigated by the broad inclusion of multiple institutions and disciplines, offering a well-rounded snapshot of current perceptions and a baseline for future longitudinal studies. To address potential bias inherent in self-reported data, the survey instrument was validated, responses were collected anonymously, and qualitative findings were used to triangulate and enrich interpretation. Although the study focused exclusively on Egyptian institutions, the diversity among participating institution types and the use of internationally recognized COHPE determinants lend credibility to the broader relevance of the findings. Also, the online survey was disseminated through institutional WhatsApp groups, which, while efficient, may have excluded faculty who are less active on these platforms or those with limited access to digital tools. Altogether, the concurrent mixed-methods approach, rigorous validation procedures, and extensive institutional representation reinforce the overall strength of the study and support confidence in the validity and reliability of its results.

## Supplementary Information

Supplementary Material 1.

Supplementary Material 2.

Supplementary Material 3.

## Data Availability

In line with the journal’s commitment to transparency, certain materials, specifically the questionnaire and topic guide, are made available as appendices or supplementary files [Appendix 1 and 2, respectively]. However, the raw records, interview transcripts, and the institutions’ affiliations of the participants in the focus group discussions cannot be made publicly available. This restriction is in place to honour the confidentiality assurances given to participants at the time of consent.

## Abbreviations

COHPE: Community-Oriented Health Professions Education
FGDs: Focus Group Discussions
CVI: Content Validity Index
ICC: Intra-Class Correlation
IRB: Institutional Review Board
FDBSU-REC: Faculty of Dentistry, Beni Suef University- Research Ethics Committee
AI: Agreement Index
D: Determinant
CFIR: Consolidated Framework for Implementation Research
PAHO: Pan American Health Organization
