# Supplementary material for: Assessment of faculty members’ perceptions towards community-oriented health professions education in Egypt: a concurrent convergent mixed-methods study
Source: BMC Med Educ. 2026 May 25;26:789. doi: 10.1186/s12909-026-09430-1 (PMC13200390; doi:10.1186/s12909-026-09430-1)
Supplement: Supplementary file 1 — Supplementary Material 1. [file 12909_2026_9430_MOESM1_ESM.pdf]

Survey on Faculty Perception of Community-Oriented Health Professions Education (COHPE)

Introduction:
This survey seeks to gather your perceptions of Community-Oriented Health Professions Education (COHPE) as implemented at your institution. Your responses will help identify areas of strength and opportunities for improvement. Please respond to each question based on your experience.

Please note that your responses will be treated in strict confidence. No information that could identify you personally or your institution will be disclosed in any reports, publications, or presentations resulting from this study. Data will be stored securely and accessed only by the research team.

Participation is voluntary, and you may choose to skip any question or withdraw from the study at any time without penalty.

By completing this questionnaire, you consent to the use of your anonymized responses for research purposes.

Operational Definitions:

1. **Community-Oriented Education (COE):**
   Community-oriented education refers to a curriculum that is designed to address the health needs and priorities of the local community. It focuses on preparing students to work effectively in community settings, emphasizing the integration of community health needs into their education and clinical practice.
2. **Community Empowerment:**
   Community empowerment involves engaging and enabling local communities to take control of their health by involving them in the decision-making, planning, and execution of health initiatives. Empowerment focuses on building the community's capacity to identify and solve its health-related issues.
3. **Social Accountability:**
   Social accountability in health professions education is the responsibility of educational institutions to direct their activities towards addressing the priority health needs of the community they serve. This includes aligning educational, research, and service efforts with the health priorities of the local population.
4. **Cultural Sensitivity:**
   Cultural sensitivity refers to the ability of healthcare providers and students to be aware of and respect cultural differences when interacting with patients from diverse backgrounds. It involves understanding and addressing cultural beliefs, practices, and language that may impact healthcare delivery.
5. **Cultural Safety:**
   Cultural safety goes beyond cultural sensitivity by addressing power imbalances and systemic inequities in healthcare. It ensures that healthcare environments are safe and respectful for individuals from different cultural backgrounds, with an emphasis on minimizing the risk of harm related to cultural insensitivity or discrimination.
6. **Health Systems Science (HSS):**
   Health systems science is the study of how healthcare is delivered, including the

understanding of healthcare systems, healthcare policies, teamwork, quality improvement, and patient safety. It equips students with the knowledge needed to navigate and improve the broader healthcare system, including community-based health services.

Please keep these definitions in mind as you complete the survey.

Demographic Information

Please provide the following information. Your responses will remain confidential and will only be used for research purposes.

1. **Age:**
    - o Under 30
    - o 30–39
    - o 40–49
    - o 50–59
    - o 60 or older
2. **Gender:**
    - o Male
    - o Female
3. **Institution Name:**

    (Please specify the institution where you are currently employed)

    - o **[Open text box]**
4. **Institution Category:**
    - o Governmental
    - o Non-governmental
5. **Faculty:**
    - o Medicine
    - o Dentistry
    - o Pharmacy
    - o Nursing
    - o Physical Therapy
6. **Academic Rank:**
    - o Lecturer

- Assistant Professor
- Professor

7. **Years of Experience in Teaching:**
    - Less than 5 years
    - 5–10 years
    - 11–15 years
    - More than 15 years
8. **Involvement in Community-Oriented Health Professions Education (COHPE):**
    - Not involved
    - Somewhat involved
    - Moderately involved
    - Very involved
    - Completely involved
9. **Have you received any formal training or education in community-oriented health professions education?**
    - Yes
    - No

Section 1: Relevance to Community Needs

1. How effectively does the curriculum address the health needs of the local community?
    - Not at all / Slightly / Moderately / Very / Extremely/I don't know
2. To what extent do faculty members participate in assessing community health needs when designing the curriculum?
    - Not at all / Slightly / Moderately / Very / Completely/I don't know

Section 2: Priority Health Problems

1. How well does the curriculum prioritize the major health problems faced by the local community?
    - Not at all / Slightly / Moderately / Very / Extremely/I don't know
2. To what extent are students trained to address the most common health challenges in the community?
    - Not at all / Slightly / Moderately / Very / Completely/I don't know

Section 3: Level of Integration of Community Orientation

1. How well is community-oriented education integrated across various parts of the curriculum?
    - Not at all / Slightly / Moderately / Very / Completely/I don't know
2. How often are students provided with opportunities to apply their knowledge in community settings?
    - Never / Rarely / Sometimes / Often / Always/I don't know

Section 4: Community Empowerment and Engagement

1. How involved are community members in the curriculum development and educational processes?
    - Not involved / Slightly involved / Moderately involved / Very involved / Completely involved/I don't know
2. How often do students actively engage with community stakeholders in their training?
    - Never / Rarely / Sometimes / Often / Always/I don't know

Section 5: Cultural Sensitivity and Safety

1. How well does the curriculum prepare students to be culturally sensitive in their clinical practice?
    - Not at all / Slightly / Moderately / Very / Extremely/I don't know
2. To what extent are faculty members trained to teach cultural competence and safety?
    - Not at all / Slightly / Moderately / Very / Completely/I don't know

Section 6: Social Accountability

1. How well does the curriculum promote social accountability by addressing the social determinants of health?
    - Not at all / Slightly / Moderately / Very / Extremely/I don't know
2. To what extent are students encouraged to contribute to improving health outcomes in the community?
    - Not at all / Slightly / Moderately / Very / Completely/I don't know

Section 7: Incorporation of Health Systems Science

1. How effectively is health systems science integrated into the curriculum to help students understand the broader healthcare system?
    - Not at all / Slightly / Moderately / Very / Extremely/I don't know
2. How often does the curriculum provide students with practical knowledge about how health systems function at the community level?
    - Never / Rarely / Sometimes / Often / Always/I don't know

Section 8: Partnering with Organizations and Government

1. How well does the institution collaborate with local organizations and governmental agencies to enhance community-oriented education?
    - Not at all / Slightly / Moderately / Very / Extremely/I don't know
2. How effective are these partnerships in enhancing students' learning experiences in community health?
    - Not effective / Slightly effective / Moderately effective / Very effective / Extremely effective/I don't know

Section 9: Community Involvement in COHPE

1. How effectively does the institution build trust with the local community before involving them in educational activities?
    - Not at all / Slightly / Moderately / Very / Completely/I don't know
2. How regularly is community diagnosis conducted to understand and address the health needs of the local population?
    - Never / Rarely / Sometimes / Often / Always/I don't know
3. How actively does the institution involve community members in decision-making processes related to health education?
    - Not at all / Slightly / Moderately / Very / Completely/I don't know
4. How well do partnerships between the institution and local government/organizations enhance the sustainability of community-oriented education?
    - Not at all / Slightly / Moderately / Very / Completely/I don't know
