# Supplementary material for: Assessment of faculty members’ perceptions towards community-oriented health professions education in Egypt: a concurrent convergent mixed-methods study
Source: BMC Med Educ. 2026 May 25;26:789. doi: 10.1186/s12909-026-09430-1 (PMC13200390; doi:10.1186/s12909-026-09430-1)
Supplement: Supplementary file 2 — Supplementary Material 2. [file 12909_2026_9430_MOESM2_ESM.pdf]

Facilitator's Focus Group Discussion (FGD) Guide

Exploring Determinants of Community-Oriented Health Professions Education (COHPE)

Introduction for Facilitator to Read Aloud

Thank you for agreeing to participate in this discussion. Your views are highly valuable and will contribute to improving understanding of how medical curricula can better address community needs.

Please note:

- Your responses will be treated in **strict confidence**.
- No personal identifiers, institutional affiliations, or other information that could reveal your identity will appear in any reports, publications, or presentations from this study.
- Audio recordings and transcripts will be stored securely and accessed only by the research team.
- Participation is voluntary, and you may choose not to answer any question or to withdraw at any point without any consequences.

By taking part in this discussion, you consent to the use of your anonymized input for research purposes.

Here are some definitions. Please put it in the back of your mind while we are conducting this interview.

1. **Community-Oriented Education (COE):**
   Community-oriented education refers to a curriculum that is designed to address the health needs and priorities of the local community. It focuses on preparing students to work effectively in community settings, emphasizing the integration of community health needs into their education and clinical practice.
2. **Community Empowerment:**
   Community empowerment involves engaging and enabling local communities to take control of their health by involving them in the decision-making, planning, and execution of health initiatives. Empowerment focuses on building the community's capacity to identify and solve its health-related issues.
3. **Social Accountability:**
   Social accountability in health professions education is the responsibility of educational institutions to direct their activities towards addressing the priority health needs of the community they serve. This includes aligning educational, research, and service efforts with the health priorities of the local population.
4. **Cultural Sensitivity:**
   Cultural sensitivity refers to the ability of healthcare providers and students to be aware of and respect cultural differences when interacting with patients from diverse backgrounds. It

involves understanding and addressing cultural beliefs, practices, and language that may impact healthcare delivery.

5. **Cultural Safety:**
   Cultural safety goes beyond cultural sensitivity by addressing power imbalances and systemic inequities in healthcare. It ensures that healthcare environments are safe and respectful for individuals from different cultural backgrounds, with an emphasis on minimizing the risk of harm related to cultural insensitivity or discrimination.

6. **Health Systems Science (HSS):**
   Health systems science is the study of how healthcare is delivered, including the understanding of healthcare systems, healthcare policies, teamwork, quality improvement, and patient safety. It equips students with the knowledge needed to navigate and improve the broader healthcare system, including community-based health services.

Warm-up Question

1. Could you briefly share your role and experience in medical or health professions education?
   - **Probes:**
     - How long have you been involved in teaching or curriculum development?
     - Have you been part of any community-based teaching initiatives?

Discussion Sections (Aligned with COHPE Determinants)

1. Relevance to Community Needs

- Main Question: How well do you feel the curriculum addresses the health needs of the communities it serves?
- **Probes:**
  - How do you find out about the health needs of the local community?
  - Are there any formal mechanisms for updating curriculum content based on emerging needs?
  - What are the main barriers to aligning teaching content with community health priorities?

2. Priority Health Problems

- Main Question: How are priority community health problems identified in your institution?
- **Probes:**
  - Who is involved in determining these priorities?
  - Are community members part of this process?
  - Can you give an example of a priority health problem and how it was incorporated into teaching?
  - What challenges do you face in keeping these priorities updated?

3. Level of Integration of Community Orientation

- Main Question: How is community orientation embedded across different parts of the curriculum?
- **Probes:**
  - Is it present in both basic and clinical sciences?
  - How early do students get exposed to community settings?
  - Are there examples of integration across multiple modules or courses?
  - What factors help or hinder this integration?

4. Community Empowerment and Engagement

- Main Question: How are communities engaged in curriculum design, delivery, and evaluation?
- **Probes:**
  - Can you share examples where community members contributed to teaching or assessment?
  - How do you maintain ongoing relationships with community partners?
  - What barriers have you faced in engaging communities meaningfully?

5. Cultural Sensitivity and Safety

- Main Question: How does the curriculum ensure cultural sensitivity and safety in teaching and learning?
- **Probes:**
  - Are there opportunities for students to learn directly from diverse cultural groups?
  - How do you address issues of cultural safety beyond just awareness?
  - What training is given to faculty in this area?

6. Social Accountability

- Main Question: In what ways does your institution demonstrate social accountability in education and service?
- **Probes:**
  - How is social accountability reflected in the institution’s mission or policies?
  - How do you measure whether you are meeting community health needs?
  - Are students involved in socially accountable projects or initiatives?

7. Incorporation of Health Systems Science (HSS)

- Main Question: To what extent are topics like health policy, teamwork, quality improvement, and systems thinking included in the curriculum?
- **Probes:**
    - Can you give examples of courses or activities that integrate HSS concepts?
    - How do students apply systems thinking in community contexts?
    - What gaps exist in covering these topics?

8. Partnerships with Organizations and Government

- Main Question: What collaborations exist between your institution and local health authorities, NGOs, or community-based organizations?
- **Probes:**
    - How do these partnerships influence curriculum design or delivery?
    - Are students given opportunities to learn in these partner settings?
    - What makes these partnerships strong or weak?

Closing Questions

- From your perspective, what are the top three priorities for strengthening COHPE in your institution?
- Is there anything we haven’t asked that you think is important to discuss?

Thank participants again for their contributions and remind them of the confidentiality of their input.
