# Supplementary material for: Assessment of faculty members’ perceptions towards community-oriented health professions education in Egypt: a concurrent convergent mixed-methods study
Source: BMC Med Educ. 2026 May 25;26:789. doi: 10.1186/s12909-026-09430-1 (PMC13200390; doi:10.1186/s12909-026-09430-1)
Supplement: Supplementary file 3 — Supplementary Material 3. [file 12909_2026_9430_MOESM3_ESM.pdf]

AFCM_Faculty Perception of Community-Oriented Health Professions Education (COHPE) (Responses)	5 point Scale								AI out of 5
		5	4	3	2	1	0	Sum	
How effectively does the curriculum address the health needs of the local community?	No.	1	150	227	51	2	23	454	3.1
	%	0.2%	33.0%	50.0%	11.2%	0.4%	5.1%		
To what extent do faculty members participate in assessing community health needs when designing the curriculum?	No.	23	113	180	93	18	27	454	2.9
	%	5.1%	24.9%	39.6%	20.5%	4.0%	5.9%		
**Section 1: Relevance to Community Needs**	No.	24	263	407	144	20	50	908	3.0
	%	2.6%	29.0%	44.8%	15.9%	2.2%	5.5%		
How well does the curriculum prioritise the major health problems faced by the local community?	No.	17	125	216	69	6	21	454	3
	%	3.7%	27.5%	47.6%	15.2%	1.3%	4.6%		
To what extent are students trained to address the most common health challenges in the community?	No.	16	92	210	94	16	26	454	2.8
	%	3.5%	20.3%	46.3%	20.7%	3.5%	5.7%		
**Section 2: Priority Health Problems**	No.	33	217	426	163	22	47	908	2.9
	%	3.6%	23.9%	46.9%	18.0%	2.4%	5.2%		
How well is community-orientated education integrated across various parts of the curriculum?	No.	18	86	211	96	15	28	454	2.8
	%	4.0%	18.9%	46.5%	21.1%	3.3%	6.2%		
How often are students provided with opportunities to apply their knowledge in community settings?	No.	43	99	203	65	17	27	454	3
	%	9.5%	21.8%	44.7%	14.3%	3.7%	5.9%		
**Section 3: Level of Integration of Community Orientation**	No.	61	185	414	161	32	55	908	2.9
	%	6.7%	20.4%	45.6%	17.7%	3.5%	6.1%		
How involved are community members in the curriculum development and educational processes?	No.	7	41	150	119	97	40	454	2.2
	%	1.5%	9.0%	33.0%	26.2%	21.4%	8.8%		
How often do students actively engage with community stakeholders in their training?	No.	37	75	191	79	36	36	454	2.8
	%	8.1%	16.5%	42.1%	17.4%	7.9%	7.9%		
**Section 4: Community Empowerment and Engagement**	No.	44	116	341	198	133	76	908	2.5
	%	4.8%	12.8%	37.6%	21.8%	14.6%	8.4%		
How well does the curriculum prepare students to be culturally sensitive in their clinical practice?	No.	36	121	187	74	13	23	454	3.1
	%	7.9%	26.7%	41.2%	16.3%	2.9%	5.1%		
To what extent are faculty members trained to teach cultural competence and safety?	No.	25	86	194	98	31	20	454	2.8
	%	5.5%	18.9%	42.7%	21.6%	6.8%	4.4%		
**Section 5: Cultural Sensitivity and Safety**	No.	61	207	381	172	44	43	908	2.9
	%	6.7%	22.8%	42.0%	18.9%	4.8%	4.7%		
How well does the curriculum promote social accountability by addressing the social determinants of health?	No.	21	90	208	83	18	34	454	2.8
	%	4.6%	19.8%	45.8%	18.3%	4.0%	7.5%		
To what extent are students encouraged to contribute to improving health outcomes in the community?	No.	33	87	188	100	19	27	454	2.9
	%	7.3%	19.2%	41.4%	22.0%	4.2%	5.9%		
**Section 6: Social Accountability**	No.	54	177	396	183	37	61	908	2.8
	%	5.9%	19.5%	43.6%	20.2%	4.1%	6.7%		
How effectively is health systems science integrated into the curriculum to help students understand the broader healthcare system?	No.	31	82	195	98	22	26	454	2.8
	%	6.8%	18.1%	43.0%	21.6%	4.8%	5.7%		
How often does the curriculum provide students with practical knowledge about how health systems function at the community level?	No.	56	97	199	55	19	28	454	3.1
	%	12.3%	21.4%	43.8%	12.1%	4.2%	6.2%		
**Section 7: Incorporation of Health Systems Science**	No.	87	179	394	153	41	54	908	2.95
	%	9.6%	19.7%	43.4%	16.9%	4.5%	5.9%		
How well does the institution collaborate with local organisations and governmental agencies to enhance community-orientated education?	No.	28	94	188	85	16	43	454	2.8
	%	6.2%	20.7%	41.4%	18.7%	3.5%	9.5%		
How effective are these partnerships in enhancing students' learning experiences in community health?	No.	27	97	170	92	21	47	454	2.7
	%	5.9%	21.4%	37.4%	20.3%	4.6%	10.4%		
**Section 8: Partnering with Organizations and Government**	No.	55	191	358	177	37	90	908	2.76
	%	6.1%	21.0%	39.4%	19.5%	4.1%	9.9%		
How effectively does the institution build trust with the local community before involving them in educational activities?	No.	28	87	172	90	19	58	454	2.6
	%	6.2%	19.2%	37.9%	19.8%	4.2%	12.8%		
How regularly is community diagnosis conducted to understand and address the health needs of the local population?	No.	37	89	193	59	15	61	454	2.8
	%	8.1%	19.6%	42.5%	13.0%	3.3%	13.4%		
How actively does the institution involve community members in decision-making processes related to health education?	No.	17	76	160	103	32	66	454	2.4
	%	3.7%	16.7%	35.2%	22.7%	7.0%	14.5%		
How well do partnerships between the institution and local government/organisations enhance the sustainability of community-orientated education?	No.	35	91	170	84	16	58	454	2.7
	%	7.7%	20.0%	37.4%	18.5%	3.5%	12.8%		
**Section 9: Community Involvement in COHPE**	No.	52	167	330	187	48	124	908	2.58
	%	5.7%	18.4%	36.3%	20.6%	5.3%	13.7%		
**Overall Survey**	No.	536	1878	3812	1687	448	719	9080	2.8
	%	6%	21%	42%	19%	5%	8%		
